# Case reports of invasive mucormycosis associated neutropenic enterocolitis in leukemic children: diagnostic and treatment challenges and review of literature

**DOI:** 10.1186/s12879-021-06957-0

**Published:** 2021-12-20

**Authors:** Ali Amanati, Omid Reza Zekavat, Hamidreza Foroutan, Omidreza Azh, Ali Tadayon, Ahmad Monabati, Mohammad Hossein Anbardar, Haleh Bozorgi

**Affiliations:** 1 Professor Alborzi Clinical Microbiology Research Center, Shiraz University of Medical Sciences, Shiraz, Islamic Republic of Iran; 2 Hematology Research Center, Shiraz University of Medical Sciences, Shiraz, Islamic Republic of Iran; 3 Laparoscopy Research Center, Shiraz University of Medical Sciences, Shiraz, Iran; 4 Department of Hematopathology, Molecular Pathology and Cytogenetics, Shiraz University of Medical Sciences, Shiraz, Iran; 5 Department of Pathology, School of Medicine, Shiraz University of Medical Sciences, Shiraz, Iran

**Keywords:** Mucormycosis, Leukemia, Neutropenic enterocolitis, Febrile neutropenia, Breakthrough fungal infection, Liposomal amphotericin B, Children

## Abstract

**Background:**

Bacterial enterocolitis is one of the most common neutropenic fever complications during intensive chemotherapy. Despite aggressive antibacterial treatments, this complication usually imposes high morbidity and mortality in cancer patients. Management of bacterial neutropenic enterocolitis are well known; however, management of fungal neutropenic enterocolitis may be more challenging and needs to be investigated. Prompt diagnosis and treatment may be life-saving, especially in patients at risk of mucormycosis-associated neutropenic enterocolitis.

**Case presentation:**

We report two mucormycosis-associated neutropenic enterocolitis cases in pediatric leukemic patients receiving salvage chemotherapy for disease relapse. Both patients' clinical signs and symptoms differ from classical bacterial neutropenic enterocolitis. They were empirically treated as bacterial neutropenic enterocolitis with anti-gram-negative combination therapy. Despite broad-spectrum antimicrobial treatment, no clinical improvement was achieved, and both of them were complicated with severe abdominal pain necessitating surgical intervention. Mucormycosis is diagnosed by immunohistopathologic examination in multiple intraoperative intestinal tissue biopsies. Both patients died despite antifungal treatment with liposomal amphotericin-B and surgical intervention.

**Conclusion:**

Mucormycosis-associated neutropenic enterocolitis is one of the most unfavorable and untreatable side effects of salvage chemotherapy in leukemic children with disease relapse. This report could be of considerable insight to the clinicians and scientists who counter the enigma of fungal infections during febrile neutropenia and help to understand better diagnosis and management.

## Background

Neutropenic enterocolitis (NEC), also known as typhlitis, is a severe form of enterocolitis with an estimated incidence ranging from 1 to 10%, varied based on age and underlying in disease neutropenic patients with malignancy [1–5]. The diagnosis is based on clinical, laboratory testing, and radiological assessment [6]. Fever, abdominal pain, nausea, vomiting, and diarrhea are common signs and symptoms in affected patients [7–9].

The most associated pathogens are gram-negative bacilli, gram-positive cocci, anaerobes, and rarely fungi [6, 7, 10]. *Candida* species are the most prevalent fungus associated with NEC, while *Aspergillus* has also been reported in a few cases [11]. Herein, we present unique fatal cases of invasive mucormycosis-associated neutropenic enterocolitis (IMANEC) in two leukemic children. Despite frequently reported gastrointestinal (GI) mucormycosis, confirmed IMANEC has never been reported to date in the context of febrile neutropenia in pediatric patients with leukemia.

## Case presentation

### Case 1

A 7-year-old girl, a known case of acute myeloblastic leukemia (AML), was admitted to our center due to central nervous system (CNS) relapse for reinduction chemotherapy on 7 October 2020. Her disease was diagnosed about 2-years before admission, and she was on maintenance chemotherapy. Reinduction chemotherapy started with cytarabine (300 mg/m^2^ as loading dose day 1 and 2 and continued by 150 mg/m^2^ from day 3), Etoposide, Idarubicin (0.5 mg/kg; day 3, 5, and 7), and dexamethasone 1.5 mg/8 h (protocol BFM-2012). She also received trimethoprim/sulfamethoxazole 2.5 mg/kg twice/day three times/week to prevent *Pneumocystis jirovecii* *pneumoniae* (PJP) and antifungal prophylaxis with liposomal amphotericin-B (L-AmB) 2.5 mg/kg every other day concurrently.

After a week, her total white blood cell (WBC) count dropped to 1120/mm^3^ with absolute neutrophil count (ANC) 100/mm^3^, which continued to decrease to less than 50/mm^3^. She developed fever and abdominal pain predominantly in the right lower quadrant one week later. The diagnose of neutropenic enterocolitis (typhlitis) was made on 21 October, and she was treated with meropenem (20 mg/kg/dose/6 h) and amikacin (15 mg/kg/dose/day), and the patient's diet changed to NPO (nil per os). Abdominopelvic ultrasonographic examination revealed a circumferentially thickened cecal and ascending colon (maximum diameter 3 mm). The thickened colon walls were hyperechoic and hypervascular, surrounded by prominent lymph nodes and minimal free fluid, all together in favor of NEC. After four days, the patient's condition improved, and no thickened cecal and colon walls were detected in ultrasonographic examination (maximal thickness 1 mm). A new episode of fever developed on 31 October. Sepsis workup was done, and meropenem switched to piperacillin-tazobactam. She also complicated *Candida* mucositis (WHO-grade 3), and her port-A-Cath and peripheral blood cultures became positive with non-albicans *Candida* (time to detection 15 and 16 h, respectively). Systemic antifungal treatment started with caspofungin (70 mg/m^2^ loading dose and 50 mg/m^2^ as maintenance therapy), and the port-A-Cath was removed urgently. There was no evidence of fungal endocarditis in echocardiography. As follow patient again developed abdominal distention, bilious vomiting, and periumbilical abdominal pain. In the serial abdominopelvic ultrasonographic examination, a gradual increase in cecal and colon wall thickness was detected, and accordingly, the patient's diet changed to NPO.

She was referred to the surgical ward with suspicion of intestinal obstruction, and an exploratory laparotomy was performed. Intraoperative findings were matted small bowel with severe adhesion of omentum in the left upper quadrant with ischemic discoloration of 50 cm of small bowel after ligament Treitz along with multiple sites of yellowish discoloration of small bowel that started from ligament Treitz with skip lesions (1–5 cm diameter) separated them from each other along the 50 cm small bowel segment. Also, multiple yellowish lesions in the omentum and meso of the small bowel and transverse colon were found. Ladd's procedure (small bowel derotation) in addition to duodenal (2nd and 3rd part) and jejunal (50 cm) resection was done, and operation terminated with end-to-end duodenojejunostomy and drain insertion. The multiple biopsies were sent for pathology. L-AmB (5 mg/kg/day) was started according to the intraoperative findings suggestive of fungal infection.

**Fig. 1:**
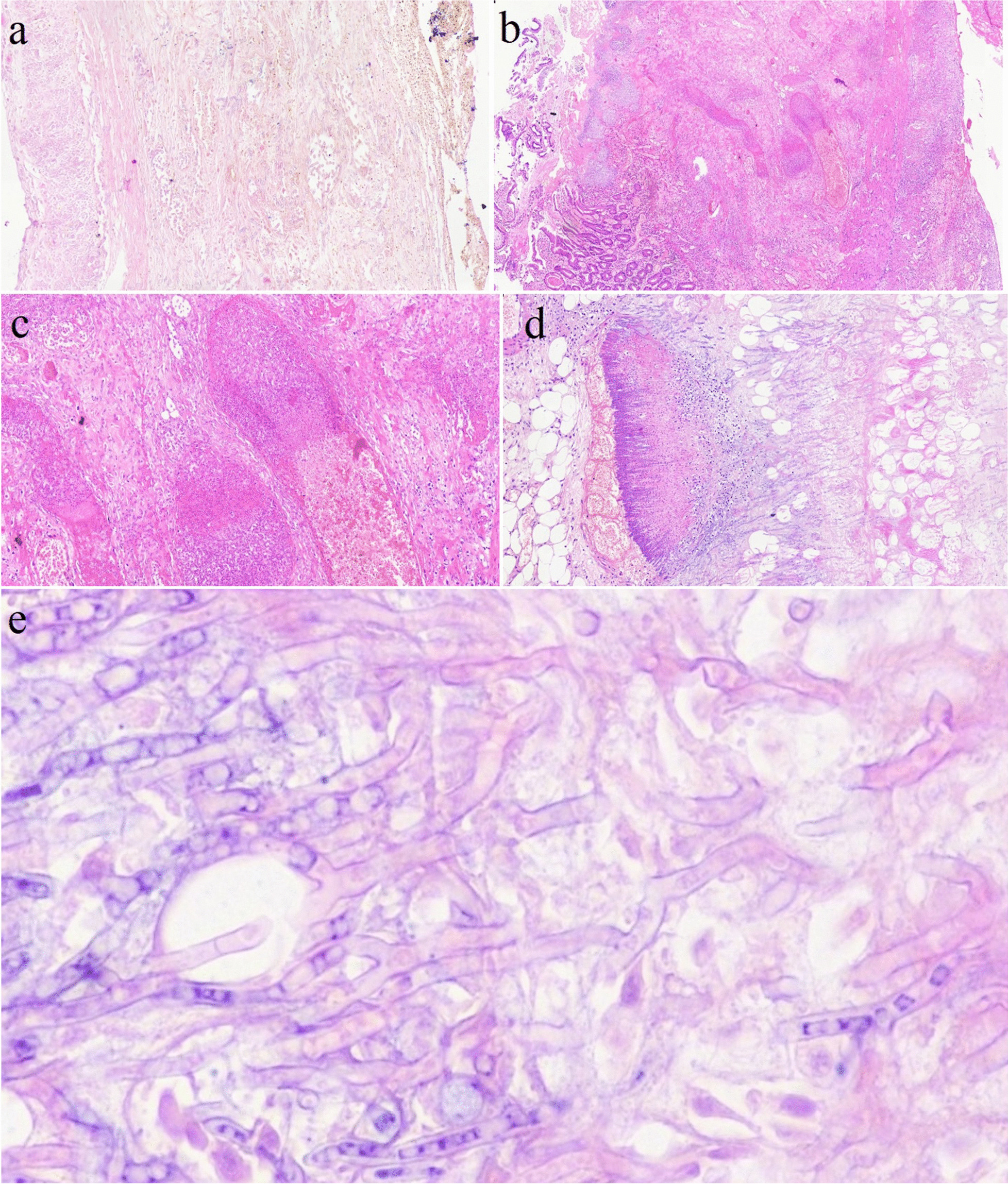
**a** The microscopic section shows small intestine transmural necrosis with extensive fungal infiltration (Hematoxylin and eosin, 200x); **b** the microscopic section from the small intestine wall shows ulceration and transmural severe acute inflammation induced by mucormycosis (Hematoxylin and eosin, 30x); **c** microscopic section shows submucosal angio-invasion by mucormycosis (Hematoxylin and eosin, 100x); **d** the microscopic section from omentum shows extensive necrosis and fungal infiltration (Hematoxylin and eosin, 100x); and **e** section shows broad, non-septate hyphae which branch irregularly compatible with mucormycosis (Hematoxylin and eosin, 700x)

After a few days, she developed disseminated intravascular coagulation (DIC). Supportive intensive care unit (ICU) care in addition to broad-spectrum antibiotic coverage including vancomycin, colistin, and metronidazole and antifungal therapy with voriconazole was continued without significant clinical improvement. Finally, she died on 16 November 2020 with multi-organ failure. Multiple intestinal biopsies showed non-septate broad hyphae in the microscopic examination by hematoxylin and eosin (H&E) staining (Fig. 1a–e). Several galactomannan tests were negative in her hospital course.

### Case 2

A 9-year-old girl with acute lymphocytic leukemia (ALL) complicated with disease relapse after about 32 months of the induction chemotherapy was admitted with pancytopenia (total WBC count 1800/mm^3^, Hb 9.6 gr/dL, and platelet 9000/ mm^3^) on 20 November 2020. She received a reinduction regimen including cytarabine (100 mg/m^2^), Etoposide, weekly vincristine, and dexamethasone 2 mg/8 h. She also received trimethoprim/sulfamethoxazole 200/40 mg twice/day three times/week for PJP prophylaxis and antifungal prophylaxis with L-AmB 2.5 mg/kg every other day concurrent with her chemotherapy. During her admission course, she was neutropenic. She developed a fever on 30 November, when her WBC count dropped to 500/mm^3^. Full sepsis workup was done, and she was put on meropenem (20 mg/kg/dose/6 h). Both port-A-Cath and blood peripheral culture became positive (time to detection 6 and 13 h, respectively) with *Klebsiella pneumoniae* sensitive to meropenem. Both cultures reported negative after 72-h of treatment.

**Fig. 2:**
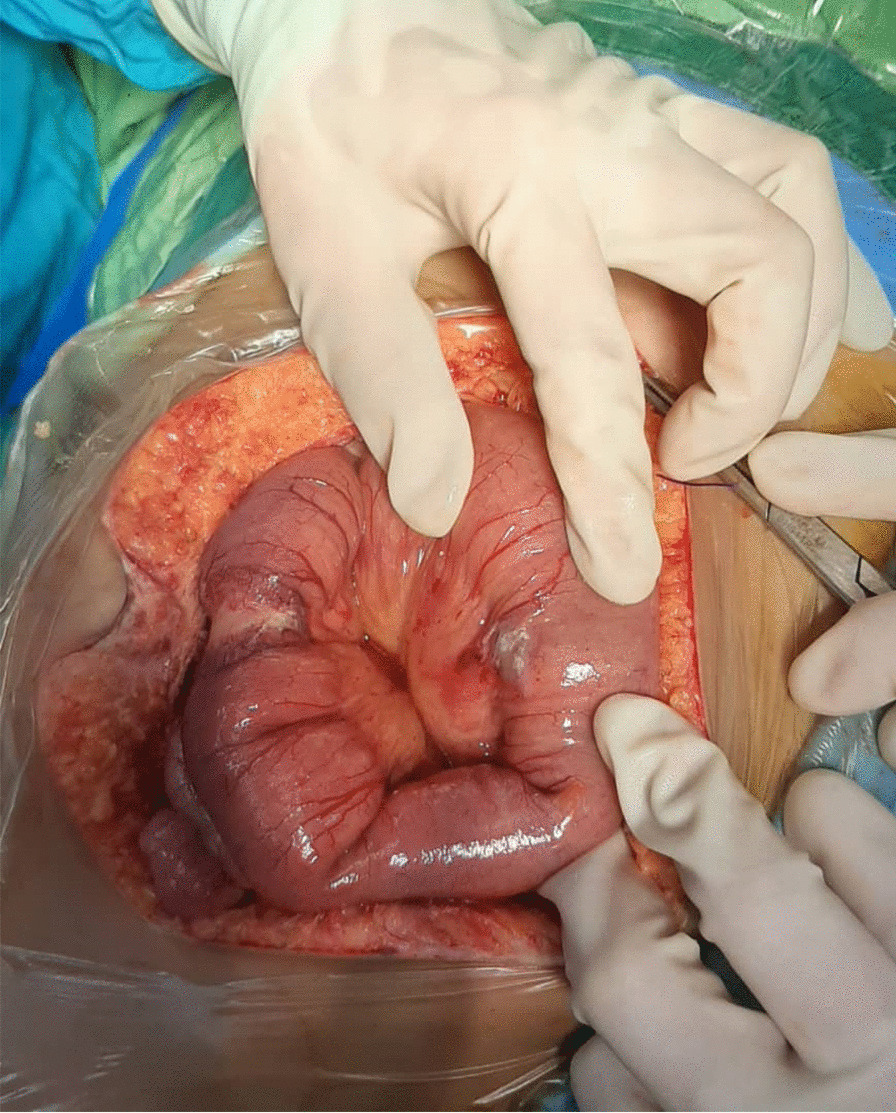
After exploratory laparotomy, an adhesion band, was found over the intestinal loop. Adhesiolysis and resection of the stenosis segment of the small bowel were done, and the operation was finished by end-to-end anastomosis

**Fig. 3:**
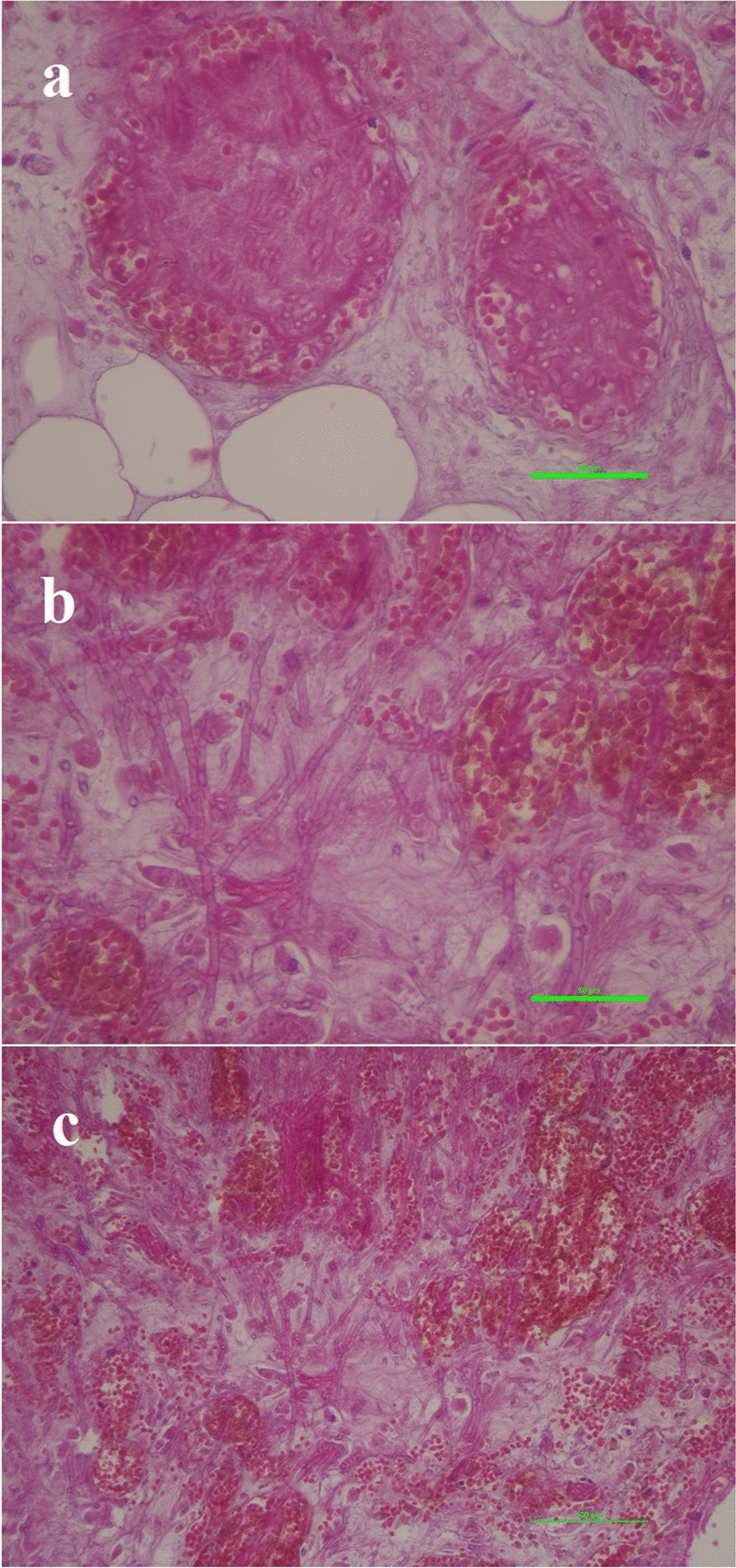
**a**–**c** Sections of omental biopsy show fungal hyphae with no clear spore formation accompanied by inflammation and tissue congestion (scale bars represents 10 µm, 50 µm, and 100 µm, respectively). H&E X400. (images created by NIS-Elements software, Nikon Instruments Inc.)

A few days later, she complicated with abdominal pain after two weeks of profound neutropenia (absolute neutrophil count < 500/mm^3^). In the abdominopelvic ultrasonographic examination, the cecal wall was thickened with a maximum diameter of 2.38 mm. The cecal wall was reported hyperechoic with mild hypervascularity. NEC was diagnosed, and the patient diet changed to NPO. Due to inadequate clinical response and continuous fever despite negative blood cultures, her antibiotic regimen changed to colistin (150,000 units/kg as a loading dose then 75,000 units/kg/12 h) and amikacin (15 mg/kg/day) on 5 December. The new antibiotic regimen achieved no clinical improvement, and with L-AmB escalated to a therapeutic dose (5 mg/kg/day) after two days. Abdominopelvic ultrasonographic findings were worsened in serial examination (maximum cecal wall thickness about 3 mm). In the third study, the small intestinal loops reveal increased thickness (maximum diameter 3.4 mm) and ileocecal involvement. The patient's abdominal pain improved, and L-AmB continued, but she could not tolerate small liquid feeding. She was transferred to the pediatric surgery ward with suspicion of small bowel obstruction, suggestive of mucormycosis. Finally, she underwent exploratory laparotomy on 17 December. Post-operation findings were two lesions on 50 cm and 10 cm of ligament Treitz and adhesion band (Fig. 2). Appendectomy via double ligation method was done, and adhesiolysis and omental biopsy were taken and sent for pathology. After laparotomy, she developed DIC, which necessitated mechanical ventilation. Metronidazole (300 mg/8 h), linezolid (300 mg/12 h), and vitamin B6 (100 mg/day) were added to her antibiotic regimen, but she died on 22 December 2020 with multi-organ failure. In the pathology report, non-septate, broad hyphae were microscopic examinations of omental biopsy (Fig. 3a–c).

Mucormycosis was documented in intestinal tissue biopsies by fungal culture (Sabouraud dextrose agar; Merck, Germany), and non-septate broad hyphae were found on direct potassium hydroxide (KOH) mount examination in both patients.

History of fresh carrot juice consumption was positive in both patients just before the clinical deterioration and worsening of the abdominal pain. It should be noticed that both of them only consume fresh carrot juice during this period.

Demographic characteristics and laboratory test results of two cases of invasive mucormycosis-associated neutropenic enterocolitis are summarized in Tables 1 and 2.

**Table 1:** Summary characteristics of two cases of invasive mucormycosis-associated neutropenic enterocolitis in pediatric leukemia patients

	Case 1	Case 2
Sex	Female	Female
Age	7-year-old	9-year-old
Underlying disease	Acute myeloid leukemia	Acute lymphoblastic leukemia
Disease station	Relapse (reinduction phase)	Relapse (reinduction phase)
Primary protocol	BMF-2012	Standard risk ALL
Primary protocol date	October 2018	April 2018
Relapse protocol	BMF-2012	MSK
Duration of neutropenia	20 days	17 days
AF prophylaxis	L-AmB	L-AmB
Duration of AF prophylaxis	22 days	10 days
FQ prophylaxis	Ciprofloxacin	Not used
Duration of FQ prophylaxis	11 days	–
Primary involved site	Cecum	Cecum and ascending colon
First intestinal thickness	2.9 mm	2.38 mm
Dietary restriction period*	27 days	19 days
Blood culture	Non-albicans *Candida*	*Klebsiella pneumonia*
Concurrent *Candida* mucositis	WHO-III**	No
Empiric antimicrobial regimen	Meropenem plus amikacin	Colistin plus amikacin
History of typhlitis	Two times (December 2018)	No
History of infectious events	Perianal abscess, carbapenem-resistant gram-negative bacteremia (Acinetobacter)	No

*AF* antifungal, *FQ* fluoroquinolone

^*^Complete food and fluid withholding

^**^World Health Organization-mucositis scale

**Table 2:** Summary of laboratory test results of two cases of invasive mucormycosis-associated neutropenic enterocolitis

	Case 1	Case 2
*At the time of typhlitis diagnosis*		
WBC count (per mm^3^)	60	390
Hemoglobin (gr/dl)	9	11.7
Platelet count (per mm^3^)	80,000	93,000
Prothrombin time	–	15.3
International normalized ratio	–	1.06
Partial thromboplastin time	–	27.9
Blood urea nitrogen (mg/dL)	16	11
Serum creatinine (mg/dl)	0.62	0.71
Alanine aminotransferase (U/L)	30	69
Aspartate aminotransferase (U/L)	16	46
Serum albumin	3.8	3.4
ESR (mm/hours)	125	111
CRP (mg/dL)	23	41
On expiration date*		
WBC count (per mm^3^)	1100	500
Hemoglobin (gr/dL)	12.8	9.4
Platelet count (per mm^3^)	17,000	17,000
Prothrombin time	24.5	33.6
International normalized ratio	1.81	2.49
Partial Thromboplastin Time	59.1	39
Blood urea nitrogen (mg/dL)	14	37
Serum creatinine (mg/dL)	0.32	1.08
Alanine aminotransferase (U/L)	14	49
Aspartate aminotransferase (U/L)	23	82
Serum albumin	3.5	3.2
CRP (mg/dL)	> 150	> 150
*Final venous blood gas*		
PH	7.25	7.018
CO2 concentration (mmHg)	40.6	33
O2 concentration (mmHg)	73.9	64
HCO3 (mmol/L)	17.9	8.5
Base excess (mmol/L)	− 8.7	− 21.7

*WBC* white blood cell, *ESR* Erythrocyte Sedimentation Rate, *CRP* C-reactive protein

^*^Both patients received fresh frozen plasma, vitamin-K, and packed cell several times

## Discussion and conclusions

We provide a comprehensive review of two proven IMANEC in leukemic children with disease relapse. IMANEC is an uncommon presentation of invasive *Mucorales* infections [12], and there are very few reports of IMANEC in pediatric patients with hematological malignancies [13–15].

Vadeboncoeur et al. described a fatal small intestinal mucormycosis in a 31-month-old boy with large cell anaplastic lymphoma early after chemotherapy presented with a gangrenous ileo-ileal intussusception [16]. Sellappan et al. reported a colonic mucormycosis in a 10-year-old male child with Down's syndrome and B-precursor-ALL during induction chemotherapy with a fatal outcome [17]. Totadri et al. described a fatal small intestinal mucormycosis in a 10-year-old male child with B-precursor-ALL during the consolidation phase of chemotherapy [18].

So given the aggressive disease nature and its fatal outcome, this report could improve our knowledge regarding possible risk factors, clinical course, diagnostic and treatment challenges, and prognosis of mucormycosis-associated NEC.

The potential risk factors in our patients are summarized in Table 3. Identified risk factors for IMANEC could be categorized as host-related factors, medications, co-infections, comorbidities, and environmental exposure to fungal spores.

**Table 3:** The potential identified risk factors in two cases of invasive mucormycosis-associated neutropenic enterocolitis

*Case 1*
Disease relapse
Prolonged neutropenia
History of recurrent typhlitis and recurrent *Candida* mucositis
Antifungal prophylaxis with L-AmB
Recent history of corticosteroid treatment
History of bacteremia (recent and past positive history)
History of fungemia
Natural carrot juice consumption early after the start of oral feeding
*Case 2*
Disease relapse
Prolonged neutropenia
Antifungal prophylaxis with L-AmB
Recent history of corticosteroid treatment
History of bacteremia
Natural carrot juice consumption early after the start of oral feeding
Local intestinal ischemia (adhesion band)

*L-AmB* liposomal amphotericin-B

Host factors related to mucormycosis associated with neutropenic enterocolitis are malignancy type [19–21], disease station (relapse) [19, 21], prolonged neutropenia [12, 22], history of typhlitis, the intensity of chemotherapy-associated immunosuppression (including chemotherapy and corticosteroids) [12, 15, 23], unsolicited drug side effects (voriconazole and L-AmB prophylaxis) [23–25], coinfections [19, 26], trauma (including surgical interventions), tissue ischemia [15, 20], and contaminated food ingestion [21, 27]. Our patients have a history of fresh carrot juice consumption during the neutropenic phase. Although the patient's carrot juice samples were not accessible for us to test for fungal contamination, unpasteurized fruit juices usually have different levels of fungal contamination, including *Rhizopus* and *Mucor* [28].

### Diagnostic challenges

Invasive mucormycosis usually is diagnosed by classical signs and symptoms representing specific organ involvement (for example, invasive rhinosinusitis [12, 27], cutaneous mucormycosis [12], and orbital involvement [29]). However, the diagnosis of mucormycosis is challenging due to the lack of standard mycological tests [21, 30], including circulating antigen detection tests and standard blood polymerase chain reaction (PCR) [31]. When prepared appropriately, biological tissue samples could confirm the diagnosis by culture and immunohistopathologic examination [32]. Repeated negative serum galactomannan (GM) tests may be used to support a diagnosis by rule outing invasive aspergillosis when clinical and radiological presentations do not help differentiate such mold infections [21]. Interventional diagnostic procedures usually are challenging to use because of bleeding tendency (including thrombocytopenia and coagulopathy) and profound neutropenia in vulnerable immunocompromised patients due to the risk of bleeding and secondary bacterial infections. Exploratory laparoscopy, endoscopy, and colonoscopy are often not applicable in patients suspected of GI mucormycosis. The main barriers to performing invasive diagnostic procedures are thrombocytopenia and the significant risk of postoperative intraabdominal complications. In this context, confirmed diagnosis usually is made in post-mortem autopsy examination of involved tissues.

Despite several limitations in the diagnostic approach of fungal associated NEC, a shifting pain (from right lower quadrant to other parts of the abdomen, especially contralateral quadrants), inadequate clinical response to broad-spectrum antibiotics, progressive ultrasonographic findings (increasing wall thickness or secondary small intestinal involvement), may be helpful clues for considering unusual etiologies such as mucormycosis for NEC. Fungal-associated NEC should be considered a breakthrough invasive fungal infection (bIFI) in neutropenic patients receiving antifungal prophylaxis such as L-AmB.

### Treatment

The recommended strategy for treating invasive mucormycosis infections consists of anti-*Mucorales* antifungals, surgical debridement, and correcting predisposing risk factors [32]. Granulocyte colony-stimulating factor (G-CSF) and granulocyte–macrophage colony-stimulating factor (GMCSF) are commonly used for correct neutropenia [21]. Among available antifungals, L-AmB is one of the best anti-*Mucorales* due to lower nephrotoxicity and the possibility of administering higher drug doses, which could deliver higher cumulative drug concentrations in a shorter time [15]. However, in patients with breakthrough mucormycosis receiving L-AmB prophylaxis new azoles should be replaced, and antifungal class should be changed [15]. Posaconazole and isavuconazole are recommended antifungals [15, 33], but such formulations are usually not available in most medical, especially in developing countries.

### Breakthrough invasive fungal infection

Although there is evidence that intermittent doses of L-AmB could prevent invasive fungal infections in immunocompromised hosts, some critical points should be considered. First, the effective prophylactic treatment with L-AmB is usually achieved by high-dose intermittent intravenous injections [34–36], so the similar effect may not be obtained by lower doses as recommended by current guidelines for prophylaxis treatment [37, 38], which necessitates further investigation for determining optimal dosage and interval [39]. Second, the accumulation of the L-AmB varies according to the different body tissues during treatment [40]. Currently, intestinal tissue drug concentrations and the prophylactic effects of L-AmB for preventing invasive intestinal fungal infection are not evident. Third, the L-AmB accumulates inside the macrophage, and its effect diminishes when the fungus is invading and interacting extracellular matrix [41]. So, in food contamination, the prophylactic effects of L-AmB may be suboptimal compared with hematogenous intestinal involvement. Finally, the type of invasive fungus may predict the prophylactic effects of L-AmB. Although successful prophylaxis treatment with L-AmB is expected for many fungus pathogens, similar effects for preventing invasive intestinal mucormycosis need to be investigated [42]. Altogether, mucormycosis-associated neutropenic enterocolitis may be considered a rare type of the bIFI in patients receiving L-AmB prophylaxis, as we described in our previous reports in cancer patients [43].

### Prognosis and outcome

Delayed proper antifungal therapy is the main prognostic factor in patients with invasive mucormycosis and substantially increased mortality [30, 44]. Besides, as documented by many researchers, antifungal therapy is usually accompanied by high mortality in invasive mucormycosis [32, 44]. Most of the reported surgical interventions are performed in patients with sino-orbital and cutaneous mucormycosis [32]. The prognosis of the GI mucormycosis and complicated NEC remained unknown due to small reported cases. As described earlier, IMANEC patients encountered several barriers to timely surgical interventions, so even timely antifungal treatment may lead to unfavorable outcomes in most cases.

In conclusion, it should be noticed that although a presumptive diagnosis of IMANEC usually is difficult, repeated negative GM tests, atypical predominant abdominal point tenderness or shifting pains, and inadequate clinical and ultrasonographic response may be helpful clues for considering unusual etiologies such as mucormycosis for NEC. Disease relapse, L-AmB prophylaxis, prolonged neutropenia, bacterial and fungal co-infections, natural fruit juice consumption may be potential risk factors for IMANEC in pediatric patients with leukemia.

## Data Availability

All data generated or analyzed during this study are included in this published article.

## Abbreviations

NEC: Neutropenic enterocolitis
IMANEC: Invasive mucormycosis-associated neutropenic enterocolitis
GI: Gastrointestinal
AML: Acute myeloblastic leukemia
CNS: Central nervous system
PJP: *Pneumocystis jirovecii* *pneumoniae*
L-AmB: Liposomal amphotericin-B
WBC: White blood cell
ANC: Absolute neutrophil count
DIC: Disseminated intravascular coagulation
ICU: Intensive care unit
ALL: Acute lymphocytic leukemia
PCR: Polymerase chain reaction
GM: Serum galactomannan tests
bIFI: Breakthrough invasive fungal infection
G-CSF: Granulocyte colony-stimulating factor
GMCSF: Granulocyte–macrophage colony-stimulating factor
ESR: Erythrocyte sedimentation rate
CRP: C-Reactive protein
